# A Rare Case of Conservative Management of Multiple Aerodigestive Fistulas in a Patient

**DOI:** 10.7759/cureus.44336

**Published:** 2023-08-29

**Authors:** Abhishek Yadav, Vivek Saini, Narendra Bhargava, Rajendra Bhati, Bobby Mitrolia

**Affiliations:** 1 Gastroenterology and Hepatology, Dr. Sampurnanand Medical College, Jodhpur, IND; 2 Medicine, Maulana Azad Medical College and Lok Nayak Hospital, Delhi, IND; 3 Gastroenterology, Dr. Sampurnanand Medical College, Jodhpur, IND; 4 Internal Medicine, Lady Hardinge Medical College and Dr. Ram Manohar Lohia Hospital, Delhi, IND; 5 Medicine, University College of Medical Sciences, Delhi, IND

**Keywords:** endoscopic clip, aero-digestive tract, gastroenterology and endoscopy, adult tracheo-esophageal fistula, anti tubercular therapy (att)

## Abstract

Acquired aerodigestive fistulas include tracheoesophageal fistulas (TEF) and bronchoesophageal fistulas (BEF). Common causes of acquired fistulas are usually malignant in origin. Tubercular tracheoesophageal fistula and bronchoesophageal fistula are rare. The limited availability of literature often presents a challenge in the treatment of tubercular TEF. We present the case of a 47-year-old woman who presented with complaints of progressive dysphagia and epigastric pain. Preliminary investigation showed raised erythrocyte sedimentation rate (ESR) of 65 mm/h and further evaluation by esophagogastroduodenoscopy for dysphagia revealed multiple ulcerated lesions in the esophagus, computed tomography (CT) revealed the presence of tracheoesophageal and bronchoesophageal fistulas with lung consolidation, and histological examination revealed granulomatous inflammation. The symptoms were managed conservatively with anti-tubercular medicine alone and showed good response.

## Introduction

Acquired aerodigestive fistulas are usually malignant in nature, commonly caused by esophageal and lung carcinomas, and rarely due to malignancies of the thyroid and larynx [1,2]. Benign acquired aerodigestive fistula has been described with long-term intubation, direct trauma to the esophagus, iatrogenic and radiation injury, post-corrosive injury, human immunodeficiency virus (HIV) infection, and chronic granulomatous lesions like tuberculosis [1,2]. Tubercular infection of the esophagus can clinically present as ulceration, stenosis, and, in rare cases, fistula formation [3,4]. Tubercular tracheoesophageal fistula (TEF) or bronchoesophageal fistula (BEF) has been reported in few case reports and is often an extension of pulmonary tuberculosis, mediastinal lymphadenopathy, or tubercular spondylitis of the spine. We present one such case of tracheoesophageal and bronchoesophageal fistula in a patient, who was treated only with an anti-tubercular medication regimen, without any endoscopic or surgical intervention.

## Case presentation

A 47-year-old woman presented with complaints of difficulty in swallowing solid food for two months, which was progressive in nature and associated with mild, non-radiating epigastric and chest pains not associated with any feature suggestive of cardiac or pulmonary origins of chest pain. The patient also had a history of mild cough, non-productive with no diurnal variation and no relationship with food intake for two months. The patient also had an undocumented history of significant weight loss. She also reported a history of low-grade, undocumented fever for two months. No history of altered bowel habits, abdominal cramps or pain, or blood in stool was reported. Her past medical history was not significant for chronic illnesses such as diabetes mellitus, tuberculosis, radiation, or trauma, and neither was there any family history of malignancy or contact with a tuberculosis patient. Her physical examinations revealed no lymphadenopathy and respiratory system examination was also within normal limit, rest of systemic examination including the per rectal examination, were unremarkable except for a body mass index (BMI) of 19.5 kg/m2, and weight of 54 kg. On further investigation, it was found that the patient’s hemoglobin (Hb) was 11.01 gm/dl, white blood cell (WBC) count was 75OO/mm^3^, with differential counts of 62% neutrophils and 38% lymphocytes, platelet count was 332 × 103 per mm3, and her kidney and liver function tests results were within the normal range (Table 1).

**Table 1:** Baseline blood investigation of the patient. Hb = hemoglobin, DLC = differential leukocyte count, WBC = white blood cell, ESR = erythrocyte sedimentation rate, AST = aspartate aminotransferase, ALT = alanine aminotransferase, ALP = alkaline phosphatase, gm = gram, mg = milligram, dl = deciliter, IU = international unit, U = units, L = liter

Investigation	Baseline	After eight weeks	Normal range
Hb (gm/dl)	11.01	11.4	12.5–15.5 gm/dl
WBC (per mm^3^)	7500	5620	4000–11000/mm^3^
DLC	P_62_L_38_	P_70_L_30_	
Platelets (10^3^/mm^3^)	332	250	150–450 (10^3^/mm^3^)
ESR (mm/h)	65		less than 20 mm/h
Urea (mg/dl)	20	26	14–40 mg/dl
Creatinine (mg/dl)	0.9	0.85	0.80–1.40 mg/dl
Bilirubin (mg/dl)	1.0	0.72	0.1–1.2 mg/dl
AST (IU/L)	24	28	less than 37 IU/L
ALT (IU/L)	18	30	less than 40 IU/L
ALP (U/L)	134	92	70–306 U/L
Total protein (gm/dl)	7.3	7.5	6.2–8.5 gm/dl
Albumin (gm/dl)	4.1	4.2	3.5–5.3 gm/dl

**Figure 1:**
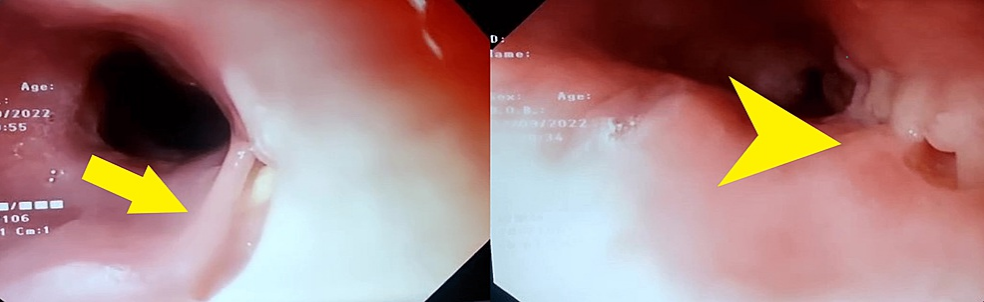
Endoscopy image at baseline Esophagogastroduodenoscopy image showing multiple openings in the esophagus at 22 cm from incisor (yellow arrow) and between 32-36cm (yellow pointed arrow) from incisors.

**Figure 2:**
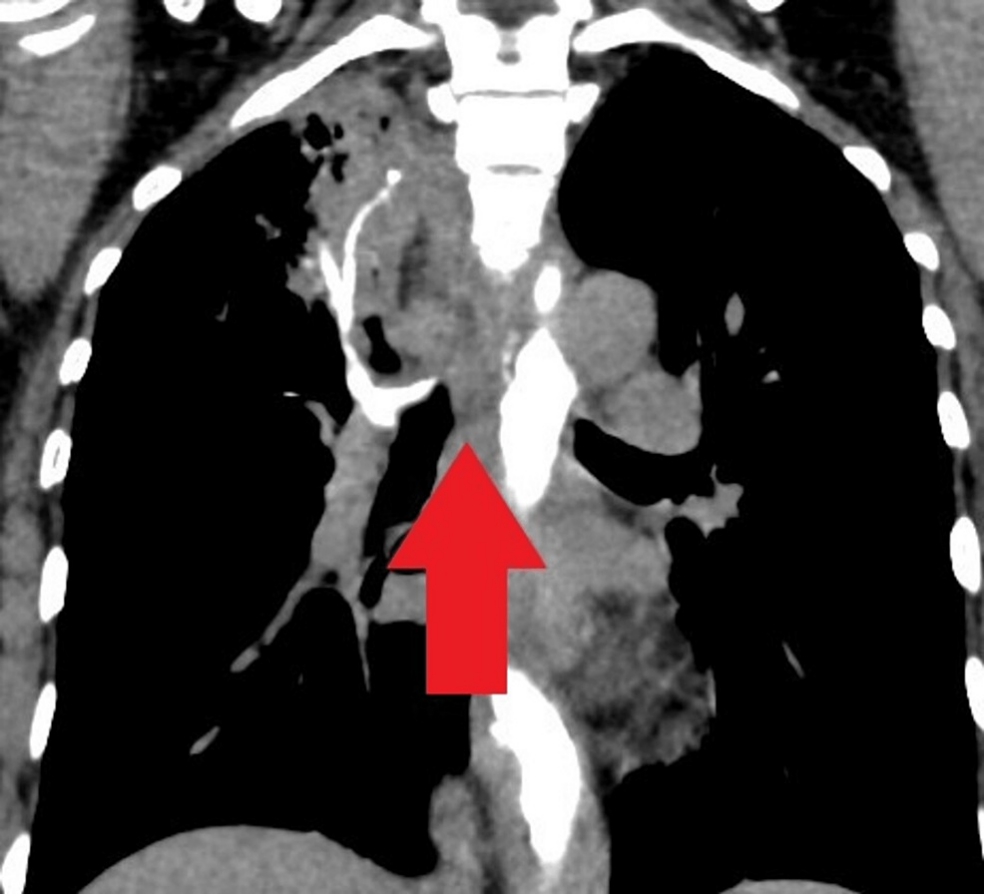
Computed tomography (CT) chest image. CT chest image red arrow pointing towards the extravasation of oral contrast into respiratory tract showing fistulous communication between esophagus and right bronchus.

**Figure 3:**
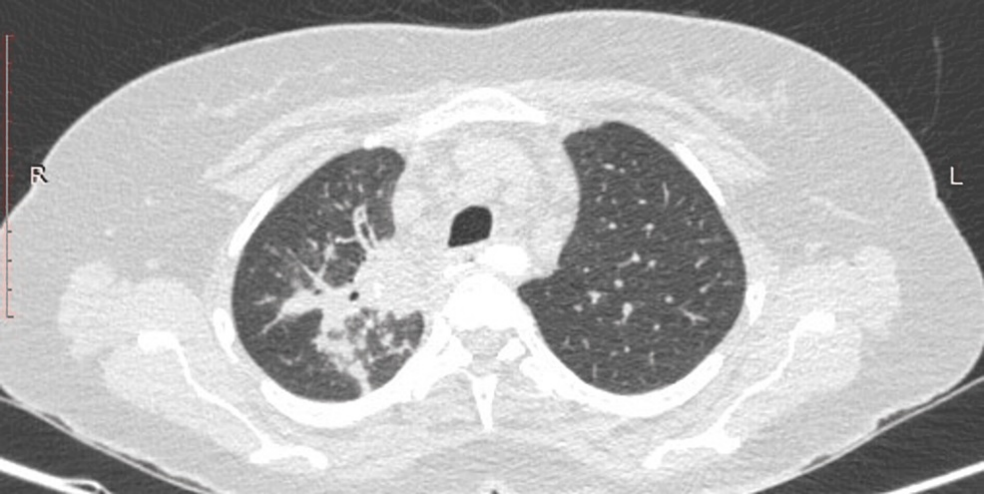
Computed tomography (CT) chest CT chest showing consolidation in right upper zone.

**Figure 4:**
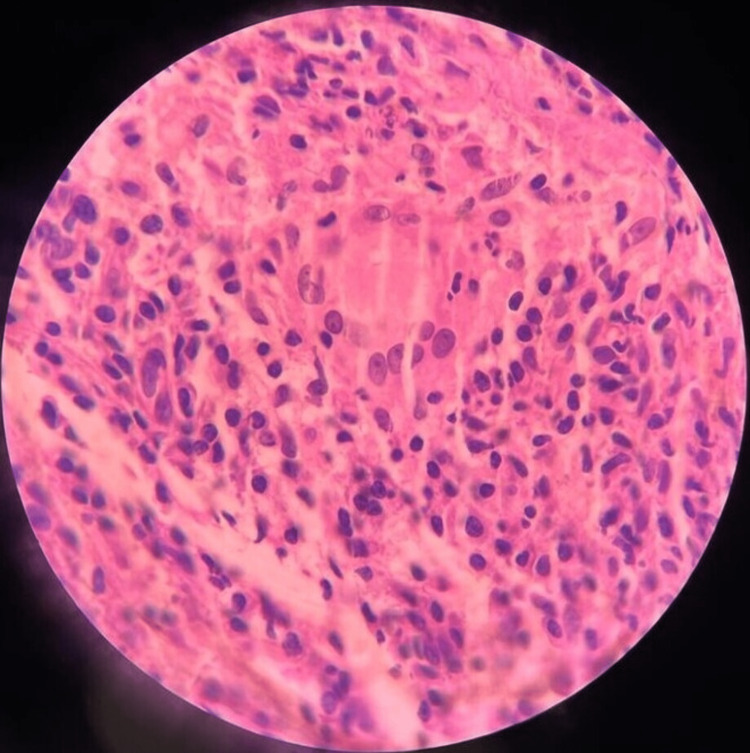
Photomicrograph of mucosal biopsy. High-power 40X H&E stain showing Langhans giant cell formation and epithelioid cell infiltrate suggestive of granulomatous inflammation.

She underwent esophagogastroduodenoscopy for the complaint of dysphagia, which revealed multiple ulcerated lesions in esophagus at 22, 32 and 36 cm from incisors (Figure 1). She also underwent computed tomography (CT) chest which revealed long segment esophageal thickening, a focal defect on the right side of the wall of the esophagus with extravasation of oral contrast into the right apical zone of the lung, another defect observed at the dependent part of the right bronchus intermediatus, indicating a bronchoesophageal fistula (Figure 2), and CT also revealed consolidation and air bronchogram in the apical and posterior segments of the right upper zone of the lung (Figure 3) and tree in bud appearance also observed at the right lower lobe of lung overall radiological finding suggestive of tubercular involvement of lung with esophagus thickening and fistulous openings. Biopsies from the ulcer margins revealed acanthotic and hyperplastic squamous epithelium, epithelioid cell aggregates, and Langhans giant cell with lymphoid infiltration finding consistent with granulomatous inflammation (Figure 4). Acid fast bacilli (AFB) stain was negative. Other examinations revealed the patient to be hepatitis B surface antigen (Hbs Ag) - negative, anti- hepatitis C virus (HCV) - negative, HIV - negative, with HbA1C of 5.8 mmol/mol, and Mantoux - positive (10 mm).

**Figure 5:**
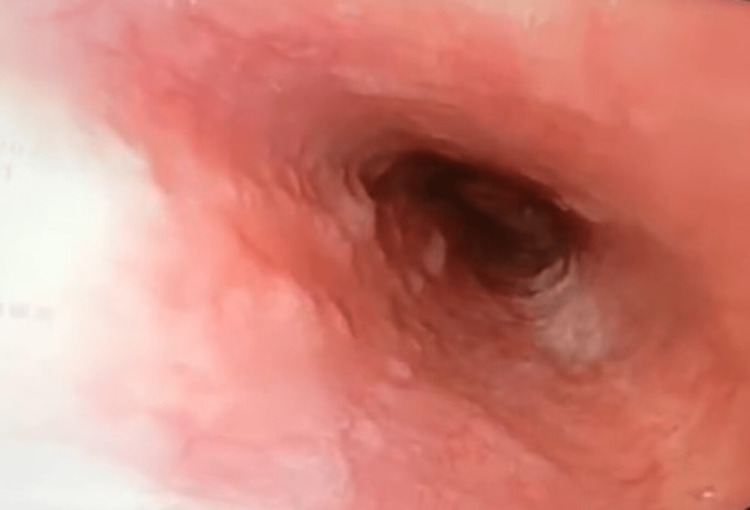
Endoscopy image after eight weeks of anti-tubercular treatment (ATT). No ulcers or fistulous opening seen.

After multidisciplinary discussion between radiologist, pathologist, internist and gastroenterologist based on clinical symptomatology and radiological findings of the tree in bud appearance with consolidation and air bronchogram in the right apical segment, and histological findings of mucosal biopsy showing epithelioid cell aggregates (granulomatous inflammation), and Langhans giant cells clinical diagnosis of tubercular consolidation with TEF and BEF was made. the patient was started on a four-drug anti-tubercular treatment (ATT) regimen consisting of isoniazid (5 mg/kg/day), rifampicin (10 mg/kg/day), ethambutol (15 mg/kg/day), and pyrazinamide (20 mg/kg/day), along with pyridoxine. The patient gradually improved symptomatically, and gained 3 kg of weight in two months. Following treatment, repeat gastroscopy conducted after eight weeks showed complete healing of the esophageal lesion (Figure 5). CT chest imaging confirmed the healing of the fistulous opening.

## Discussion

Tuberculosis continues to be a very prevalent disease in the developing countries. However, esophageal tuberculosis is exceedingly rare, accounting for roughly 2.8% of all cases [5]. While the exact prevalence of tubercular tracheoesophageal fistula and bronchoesophageal fistula remains uncertain, it is one of the rare presentations of pulmonary tuberculosis [4]. A few case reports do exist for tubercular tracheoesophageal fistula, as given in Table 2. The pathogenesis of aerodigestive fistula is usually an extension of a tubercular lesion from the lungs, mediastinal lymph nodes, or vertebra into the esophagus, rather than primary esophageal tuberculosis. Primary tuberculosis of esophagus (without any adjacent organ involvement) is rare because of the protective mechanisms involved in the esophagus like rapid clearance of ingested bacteria, tubular stricture with stratified squamous epithelium (less permeable) without any mucosal fold so no stasis of bacteria with possibly protective effect of saliva and salivary enzymes.

**Table 2:** Tubercular tracheoesophageal fistula and treatment options used. ATT = anti-tubercular treatment, PEG = percutaneous endoscopic gastrostomy, ASD = atrial septal defect. ELLA Danis stent (ELLA-CS, Hradec Králové, Czechia) is a type of retrievable self-expandable metal stent often used in uncontrollable upper gastrointestinal bleeding,

Authors	Management	No of cases
Ravi et al.(2021) [6]	ATT (duration not mentioned), plus ELLA Danis stent and surgery	01
Desai et al. (2019) [7]	ATT for 9 months and PEG tube placement	01
Gajendra et al. (2014) [8]	ATT (duration not mentioned)	01
Mutlu et al. (2019) [9]	ATT for 1 month (patient succumbed to illness) and tracheal stenting	01
Shah et al. (2022) [10]	ATT for 2 years as was case of drug resistance tuberculosis, stapler, ASD closure device, surgery	01
Khanna et al. (2017) [11]	ATT for 6 months	01
Rawat et el. (2021) [12]	ATT (duration not mentioned)	01

The clinical presentations of tracheoesophageal fistula vary depending upon the size of the fistula, site and magnitude of the leak, leading to aspiration. The patient can present with paroxysmal productive cough after eating, which does not improve despite being on ATT because of repeated episodes of aspiration, or with dysphagia, hematemesis, and constitutional symptoms like weight loss and fever [5].

Our patient with tubercular fistulas did not have clinical features of aspiration, probably because despite having multiple fistulas, the fistulous openings were smaller (less than 5 mm). Therefore, it was decided that the patient did not require primary defect closure with the help of clip, stent etc., either endoscopically or surgically, as is often described in available literature [6,9,10]. Endoscopic or surgical correction became necessary if the size of the defect is large (more than 5 mm) or patient has recurrent infection or sepsis because of aspiration. She was started on ATT in the clinical setting, supported by histological and imaging evidence. Our patient improved just on ATT and with the help of granulation tissue formation. Duration of ATT treatment is often six to nine months unless dealing with drug resistance tuberculosis where duration can be longer. This case represents a rare presentation of a common disease in the developing world, which was managed conservatively

## Conclusions

Both TEF and BEF fistula are rare complications of tuberculosis and often require multispecialty decision-making involving a gastroenterologist, pulmonologist, internal medicine specialist, and surgeons. However, anti-tubercular drugs remain the mainstay of the treatment. Smaller fistula (˂ 5 mm opening) without concerning aspiration, uncontrolled infection, or dysphagia can be managed effectively with the help of anti-tubercular drugs, while larger fistulas with repeated episodes of aspiration might require either endoscopic therapy in the form of device closure, glue, atrial septal defect (ASD) patch, clip, stenting, or surgical correction.

## References

[1] Aggarwal D, Mohapatra PR, Malhotra B (2009). Acquired bronchoesophageal fistula. Lung India.

[2] Kim HS, Khemasuwan D, Diaz-Mendoza J, Mehta AC (2020). Management of tracheo-oesophageal fistula in adults. Eur Respir Rev.

[3] Gomes J, Antunes A, Carvalho A, Duarte R (2011). Dysphagia as a manifestation of esophageal tuberculosis: a report of two cases. J Med Case Rep.

[4] Jain SK, Jain S, Jain M, Yaduvanshi A (2002). Esophageal tuberculosis: is it so rare? Report of 12 cases and review of the literature. Am J Gastroenterol.

[5] Patel N, Amarapurkar D, Agal S, Baijal R, Kulshrestha P, Pramanik S, Gupte P (2004). Gastrointestinal luminal tuberculosis: establishing the diagnosis. J Gastroenterol Hepatol.

[6] Ravi A, Kumar H, Samanta J, Mishra AK, Chauhan V (2021). Broncho-esophageal fistula following pulmonary tuberculosis and options for management. Tro Gast.

[7] Desai P, Mayenkar P, Northrup TF, Mallela V (2019). Bronchoesophageal fistula due to esophageal tuberculosis. Case Rep Infect Dis.

[8] Bhati G, Biju P, Raja K, Deepak B, Pazhanivel M (2014). Tuberculous bronchoesophageal fistula: a case report. Int J Adv Med H.

[9] Güçsav MO, Gayaf M, Aksel N, Ceylan KC, Alizoroğlu D (2019). Tracheoesophageal fistula secondary to tuberculosis: a case report. Respir Case Rep.

[10] Shah SJ, Jadhav UE, Agrawal DP (2022). Acquired tracheo-esophageal fistula in adult-a classical case of 'what not to do'. Indian J Thorac Cardiovasc Surg.

[11] Khanna V, Kumar A, Alexander N, Surendran P (2017). A case report on esophageal tuberculosis - a rare entity. Int J Surg Case Rep.

[12] Rawat A, Govil A, Agrawal D, Udawat H, Vaishnav S (2021). Esophageal tuberculosis with esophagopulmonary fistula: a case report. Ind J Case Reps.

